# Ehrlichia chaffeensis Outer Membrane Protein 1-Specific Human Antibody-Mediated Immunity Is Defined by Intracellular TRIM21-Dependent Innate Immune Activation and Extracellular Neutralization

**DOI:** 10.1128/IAI.00383-19

**Published:** 2019-11-18

**Authors:** Thangam Sudha Velayutham, Sandeep Kumar, Xiaofeng Zhang, Nurgun Kose, David H. Walker, Gary Winslow, James E. Crowe, Jere W. McBride

**Affiliations:** a Department of Pathology, University of Texas Medical Branch, Galveston, Texas, USA; b Department of Microbiology and Immunology, University of Texas Medical Branch, Galveston, Texas, USA; c Sealy Institute for Vaccine Sciences, University of Texas Medical Branch, Galveston, Texas, USA; d Center for Biodefense and Emerging Infectious Diseases, University of Texas Medical Branch, Galveston, Texas, USA; e Institute for Human Infections and Immunity, University of Texas Medical Branch, Galveston, Texas, USA; f Department of Microbiology & Immunology, Upstate Medical University, Syracuse, New York, USA; g Vanderbilt Vaccine Center, Vanderbilt University Medical Center, Nashville, Tennessee, USA; Washington State University

**Keywords:** *Ehrlichia chaffeensis*, TRIM21, antibody function, human monoclonal antibodies, neutralizing antibodies, selective autophagy

## Abstract

Antibodies are essential for immunity against Ehrlichia chaffeensis, and protective mechanisms involve blocking of ehrlichial attachment or complement and Fcγ-receptor-dependent destruction. In this study, we determined that major outer membrane protein 1 (OMP-19) hypervariable region 1 (HVR1)-specific human monoclonal antibodies (huMAbs) are protective through conventional extracellular neutralization and, more significantly, through a novel intracellular TRIM21-mediated mechanism. Addition of OMP-1-specific huMAb EHRL-15 (IgG1) prevented infection by blocking attachment/entry, a mechanism previously reported; conversely, OMP-1-specific huMAb EHRL-4 (IgG3) engaged intracellular TRIM21 and initiated an immediate innate immune response and rapid intracellular degradation of ehrlichiae. EHRL-4-TRIM21-mediated inhibition was significantly impaired in TRIM21 knockout THP-1 cells. EHRL-4 interacted with cytosolic Fc receptor TRIM21, observed by confocal microscopy and confirmed by co-immunoprecipitation. *E. chaffeensis*-EHRL-4-TRIM21 complexes caused significant upregulation of proinflammatory cytokine/chemokine transcripts and resulted in rapid (<30 min) nuclear accumulation of NF-κB and TRIM21 and ehrlichial destruction. We investigated the role of TRIM21 in the autophagic clearance of ehrlichiae in the presence of EHRL-4. Colocalization between EHRL-4-opsonized ehrlichiae, polyubiquitinated TRIM21, autophagy regulators (ULK1 and beclin 1) and effectors (LC3 and p62), and lysosome-associated membrane protein 2 (LAMP2) was observed. Moreover, autophagic flux defined by conversion of LC3I to LC3II and accumulation and degradation of p62 was detected, and EHRL-4-mediated degradation of *E. chaffeensis* was abrogated by the autophagy inhibitor 3-methyladenine. Our results demonstrate that huMAbs are capable of inhibiting *E. chaffeensis* infection by distinct effector mechanisms: extracellularly by neutralization and intracellularly by engaging TRIM21, which mediates a rapid innate immune response that mobilizes the core autophagy components, triggering localized selective autophagic degradation of ehrlichiae.

## Abstract (precis)

Antibodies are essential for immunity against Ehrlichia chaffeensis, and protective mechanisms involve blocking of ehrlichial attachment or complement and Fcγ-receptor-dependent destruction. In this study, we determined that major outer membrane protein 1 (OMP-19) hypervariable region 1 (HVR1)-specific human monoclonal antibodies (huMAbs) are protective through conventional extracellular neutralization and, more significantly, through a novel intracellular TRIM21-mediated mechanism.

## INTRODUCTION

Ehrlichia chaffeensis is a Gram-negative obligately intracellular bacterium that is transmitted by the lone star tick, Amblyomma americanum, and causes human monocytotropic ehrlichiosis (HME), one of the most prevalent life-threatening tick-borne zoonoses in the United States (1, 2). HME manifests as a life-threatening undifferentiated febrile illness with symptoms that include fever, headache, malaise, nausea, diarrhea, and cough, resulting in patient hospitalization in 43% to 62% of cases, which, if left untreated, can be fatal (3–5). From 2000 to 2017, more than 15,000 cases of HME were reported to the Centers for Disease Control, but prospective studies suggest the actual number of cases is underestimated by 100-fold (6). Clinical diagnosis of HME is difficult, and there are no vaccines available. Therapeutic options are limited to doxycycline, which is most effective when administered early in the course of infection (7, 8).

*E. chaffeensis* exhibits tropism for mononuclear phagocytes and has evolved molecular mechanisms to reprogram the phagocyte in order to survive, replicate, and effectively evade innate and adaptive immunity (9–11). Immunity to *E. chaffeensis* infection involves both humoral and cellular immune responses (12–19). Cellular immunity is primarily mediated by gamma interferon (IFN-γ)-producing antigen-specific CD4^+^ T cells (13, 17, 20), but humoral immunity contributes significantly to *E. chaffeensis* clearance, even in the absence of cell-mediated responses (12, 14, 15, 18, 21–23). Studies have demonstrated that immune serum or outer membrane protein (OMP)-specific monoclonal antibodies protect SCID mice from fatal ehrlichial infection, even when administered after infection is established (12, 14, 21). Moreover, passive transfer of epitope-specific *E. chaffeensis* tandem repeat protein (TRP) effector antisera protected mice against a lethal infection, while *in vitro* administration of antibodies both prophylactically and therapeutically inhibited infection, demonstrating potential involvement of both extracellular and intracellular antibody-mediated mechanisms (22). Humoral immunity to *E. chaffeensis* occurs, at least in part, during the extracellular stage by blocking cellular entry or attachment or via Fcγ receptor (FcγR)-dependent mechanisms (24). There is substantial evidence supporting a role for other undefined intracellular and extracellular antibody-mediated mechanisms in immunity to intracellular microbes (22), however, such as formation of immune complexes, uptake by pinocytosis/endocytosis, or engagement of intracellular Fc receptors (FcRs) such as TRIM21. The effector mechanisms and cellular context of antibody-mediated immunity to *E. chaffeensis* are not completely defined. Understanding protective immune mechanisms that control intracellular pathogens is necessary for developing effective vaccines against *Ehrlichia* spp. and other intracellular pathogens.

Tripartite motif protein 21 (TRIM21), a conserved, ubiquitously expressed, high-affinity antibody receptor in humans, was recently reported to engage in antibody-dependent intracellular neutralization (ADIN) and intracellular antibody-mediated degradation (IAMD) of several nonenveloped viruses by recruiting the proteasome and the molecular unfoldase, valosin-containing protein (VCP) (25–28). ADIN is facilitated by antibodies that fail to block entry of the pathogen into the cell or are intercepted by classical extracellular FcRs which mediate antibody-dependent cellular phagocytosis. Antibodies which escape the classical antibody-mediated mechanisms in the extracellular environment and are carried into the cell bound to the pathogen as complexes are detected by TRIM21. Detection by TRIM21 initiates rapid concurrent effector and sensor mechanisms in contrast to classical FcR-mediated sensor-then-effector immune responses. It has also been shown that antibody-coated *Salmonella* (intracellular) is sensed by TRIM21, provoking antibody-dependent NF-κB activation (27, 29). A recent study has shown the involvement of TRIM21 in the selective autophagic degradation of inflammatory signaling regulators, such as dimeric interferon regulatory factor 3 (IRF3) and active IκB kinase beta (IKKβ), which modulates gene expression of type 1 interferons and cytokines (30–32).

In the present study, we demonstrate that *E. chaffeensis* OMP-1-specific human monoclonal antibodies (huMAbs) inhibit infection through both extracellular and intracellular effector mechanisms. EHRL-15 blocked *E. chaffeensis* entry, while EHRL-4 inhibited infection by engaging the intracellular cytosolic FcR TRIM21. The engagement of the EHRL-4-*E. chaffeensis* complex was sensed by TRIM21, initiating a significant proinflammatory response and simultaneous recruitment of autophagic regulators and effectors, leading to rapid degradation of *E. chaffeensis* by selective autophagy. These findings provide a significant advancement toward understanding the molecular and cellular basis of adaptive immune responses to the obligately intracellular pathogen *E. chaffeensis* and suggest new strategies for immunotherapeutics.

## RESULTS

### Characterization of *E. chaffeensis*-specific huMAbs.

**FIG 1:**
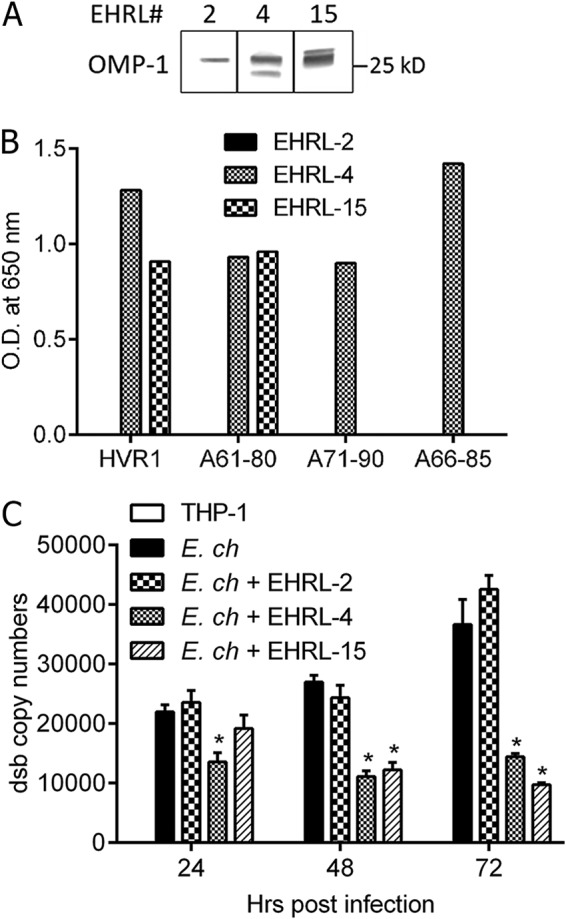
Characterization of *E. chaffeensis* OMP-1-specific huMAbs. (A) Three of the *E. chaffeensis*-specific huMAbs (EHRL-2, -4, and-15) recognized the OMP-1 (∼28 kDa) in *E. chaffeensis* whole-cell lysate by Western immunoblotting. (B) huMAb recognition of overlapping peptides within the OMP-1 HVR1 by ELISA, demonstrating fine specificity of EHRL-4 and EHRL-15. (C) The *E. chaffeensis* OMP-1-specific huMAbs tested for inhibition of ehrlichial growth as determined by *in vitro* ehrlichial inhibition assay. THP-1 cells were incubated with antibodies and then inoculated with cell-free *E. chaffeensis*; infection status was determined on days 1, 2, and 3 by qPCR. Bar graphs represent means ± SEMs. *, *P* < 0.05.

Eight *E. chaffeensis*-specific huMAbs were tested, and the majority of them recognized the outer membrane protein (OMP-1) and tandem repeat protein 32 (TRP32). This finding was determined by enzyme-linked immunosorbent assay (ELISA) and immunoblot analysis with whole *E. chaffeensis* antigenic extract, or recombinant antigens, as shown in Fig. 1A and summarized in Table 1. These results were consistent with previous studies which identified OMPs and TRPs as immunodominant determinants of protective immune responses during *E. chaffeensis* infection (33–36). Five huMAbs inhibited *E. chaffeensis* infection *in vitro* when THP-1 cells were pretreated with the huMAbs and infected with host cell-free ehrlichiae, and the bacterial load determined on day 3 postinfection (see Fig. S1A in the supplemental material). To understand the mechanisms of antibody-mediated immunity to the intracellular bacterium *E. chaffeensis*, we selected three outer membrane protein (OMP)-specific huMAbs (i.e., EHRL-2, -4, and -15), two of which demonstrated >80% inhibition of ehrlichial growth *in vitro*, and one huMAb which was nonneutralizing and that served as an internal control.

**TABLE 1:** Characterization of *E. chaffeensis* OMP-1-specific huMAbs

huMAb no.	WB^*a*^ or ELISA target	IFA result (1:500)	Inhibition (%)	IgG subclass
EHRL-2	OMP-1	+/−	0	IgG1
EHRL-4	OMP-1	+++	86	IgG3
EHRL-15	OMP-1	+++	82	IgG1

a WB, Western blotting.

All the anti-OMP-1 huMAbs recognized proteins of approximately ∼26 to 28 kDa, as determined by immunoblot analysis of whole *E. chaffeensis* antigen (Fig. 1A), which is consistent with the OMP antigens previously described for *E. chaffeensis* (36–38). In addition, EHRL-4 and EHRL-15 recognized a 30-amino-acid immunodominant peptide corresponding to the first hypervariable region (HVR1) of OMP-19 (21); the nonneutralizing EHRL-2 did not react with this peptide (Fig. 1B). Both huMAbs recognized a similar epitope within the OMP-1 HVR1.

Strong reactivity of EHRL-4 and EHRL-15 with *E. chaffeensis* was also observed by immunofluorescence assay (IFA); conversely, EHRL-2 was weakly immunoreactive (Table 1). These data indicate that protective human antibody responses represented by the huMAbs target *E. chaffeensis* OMP-1 HVR1. These and previous data (12, 21) demonstrate that both mice and humans generate protective antibodies against OMP-1 HVR1.

### *E. chaffeensis* OMP-1-specific huMAbs inhibited infection *in vitro*.

The neutralizing abilities of the OMP-1-specific huMAbs were examined using an *in vitro* ehrlichial neutralization assay. The optimal neutralizing activity of the antibodies was at 1 μg to 10 μg/ml (Fig. S1B); thus, 5 μg/ml was used for all the experiments. The neutralizing capability of the OMP-1-specific huMAbs on days 1, 2, and 3 postinfection is shown in Fig. 1C. EHRL-4 and EHRL-15 were able to inhibit *E. chaffeensis* infection *in vitro*, while EHRL-2 was ineffective.

**FIG 2:**
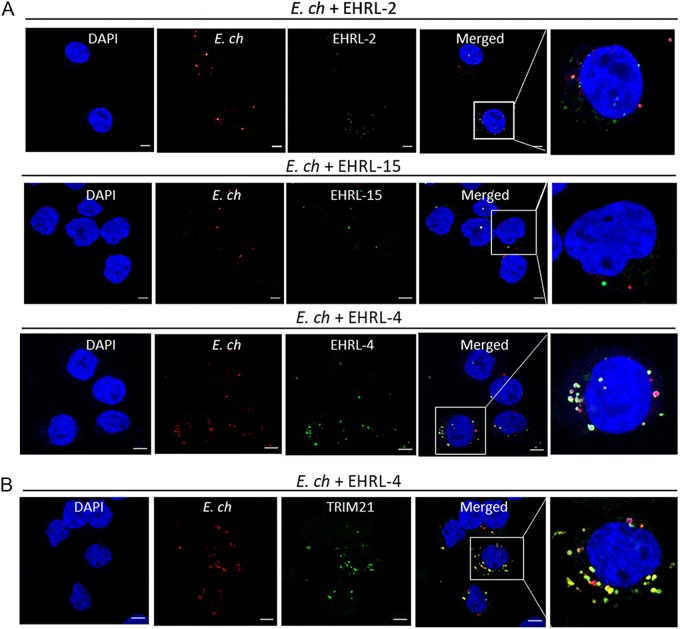
*E. chaffeensis* infection and TRIM21 colocalization in the presence of OMP-1-specific huMAb. THP-1 cells were infected with *E. chaffeensis* in the presence of anti-OMP-1 huMAbs and fixed 30 min postinfection. Confocal immunofluorescence microscopy shows internalization of anti-OMP-1 EHRL-4-bound (green) *E. chaffeensis* (red) in THP-1 cells (A) and anti-OMP-1 EHRL-4-bound *E. chaffeensis* complexes (red) colocalizing with TRIM21 (green) intracellularly (B). Cells were visualized under 63× oil immersion lens. Bars, 10 μm.

To identify mechanisms whereby anti-OMP-1 huMAbs inhibit *E. chaffeensis* infection *in vitro*, samples were collected at different time points postinfection for confocal microscopy. EHRL-4-opsonized *E. chaffeensis* was observed within the THP-1 cells (Fig. 2A) as early as 30 min postinfection, whereas, in the case of EHRL-15, significantly fewer antibody-coated ehrlichiae were detected within the cells. There were significantly more ehrlichiae internalized in samples treated with EHRL-2, but very few of them colocalized with IgG, clearly demonstrating that EHRL-2 did not bind efficiently to *E. chaffeensis* nor could it inhibit infection (Fig. 2A). These data suggest that the protective huMAbs function via different mechanisms: EHRL-15 prevented entry of *E. chaffeensis*, likely by blocking adhesion and entry, whereas EHRL-4-opsonized *E. chaffeensis* entered the cells efficiently and is likely neutralized intracellularly.

Since a large number of EHRL-4-opsonized ehrlichiae were observed within the cells, we performed a dual-staining IFA for *E. chaffeensis* and TRIM21, which is the cytosolic Fc receptor, to determine whether opsonized ehrlichiae were detected by this intracellular FcR. TRIM21 has been shown to interact with and restrict antibody-coated viruses and *Salmonella* (27). As shown in Fig. 2B, TRIM21 was efficiently recruited to the EHRL-4-coated ehrlichiae in THP-1 cells at 30 min postinfection, forming puncta, suggesting the involvement of TRIM21 in EHRL-4-mediated inhibition of *E. chaffeensis* infection. In the absence of EHRL-4, the ehrlichiae did not colocalize with endogenous TRIM21 (see Fig. S2), suggesting that TRIM21 recognized only antibody-coated ehrlichiae and not unopsonized ehrlichiae.

### Inhibition of *E. chaffeensis* infection via a TRIM21-dependent mechanism.

**FIG 3:**
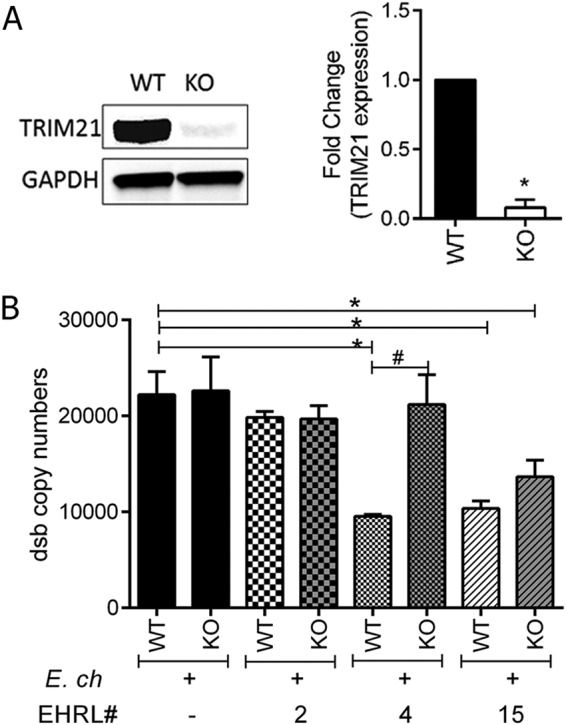
Role of TRIM21 in inhibition of *E. chaffeensis* infection by OMP-1 huMAb EHRL-4. THP-1 WT and TRIM21 KO cells were infected with *E. chaffeensis* opsonized with huMAbs (EHRL-2, -4, and -15), and ehrlichial infection was determined on day 3 postinfection by qPCR. (A) TRIM21 KO cells were generated using genome editing by CRISPR-Cas9, and the WT and KO cells were analyzed by Western blotting for expression of TRIM21 protein. (B) *E. chaffeensis* infection was determined 3 days postinfection by qPCR in WT and TRIM21 KO cells. Bar graphs represent means ± SEMs. *, *P* < 0.05 when comparing WT to WT; ^#^, *P* < 0.05 when comparing KO to WT for each huMAb.

To study how TRIM21 mediates EHRL-4-dependent *E. chaffeensis* inhibition, TRIM21 knockout (KO) THP-1 cells were developed. The TRIM21 gene in THP-1 cells was disrupted using CRISPR/Cas9 technology, cell populations with significant TRIM21 KO were selected by serial dilution, and TRIM21 protein expression was confirmed by immunoblotting (Fig. 3A). To determine if the loss of TRIM21 affected antibody-mediated neutralization, both wild-type (WT) and TRIM21 KO THP-1 cells were incubated with EHRL-4-opsonized ehrlichiae, and infection was quantitated. Infectivity of *E. chaffeensis* was similar in both the WT and TRIM21^−/−^ cells, and the EHRL-2 was nonneutralizing in both the WT and KO cells (Fig. 3B). EHRL-4 efficiently neutralized the ehrlichiae in WT cells on day 3 postinfection, and inhibitory activity was significantly reduced in the TRIM21^−/−^ cells (Fig. 3B), demonstrating that EHRL-4 inhibition was TRIM21-dependent. Infection was not affected in TRIM21 KO cells treated with EHRL-15, demonstrating that this antibody functions independent of TRIM21.

### *E. chaffeensis*-EHRL-4 complex stimulates rapid immune signaling and a proinflammatory response.

**FIG 4:**
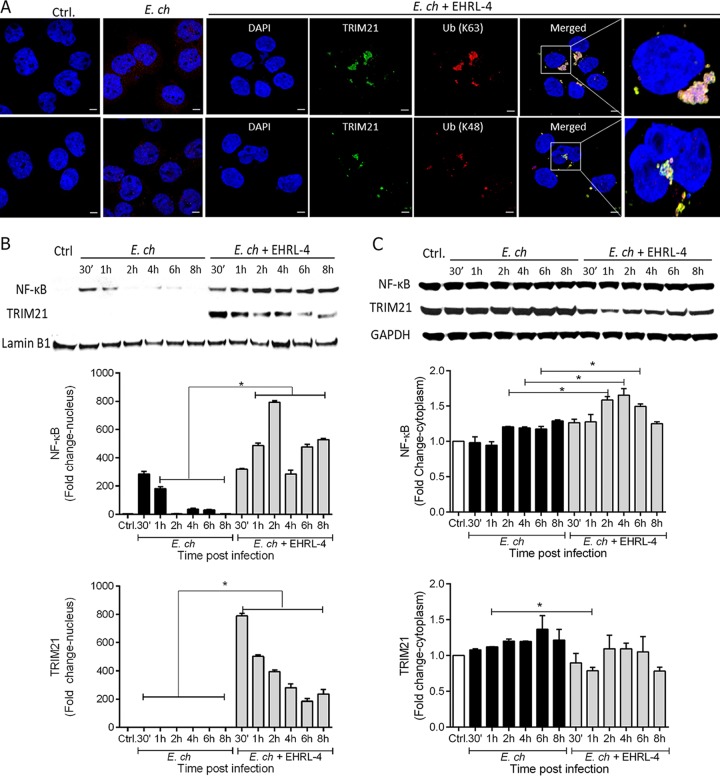
OMP-1 huMAb EHRL-4-*E. chaffeensis* complex detection by TRIM21 initiates immune signaling. THP-1 was infected with *E. chaffeensis* in the presence or absence of EHRL-4, and samples were collected for confocal microscopy and examination of cytoplasmic and nuclear fractions at various time points postinfection. (A) Confocal images show TRIM21 (green) recruited to the *E. chaffeensis*-EHRL-4 complexes colocalizing with K63- and K48-linked Ub chains (red) at 30 min postinfection. Bars, 10 μm; ×63 magnification (oil immersion lens). The control (ctrl) and *E. ch* panels represent the merged images for TRIM21 and K63/48-linked Ub chains in uninfected and infected cells. Western blot analysis of nuclear (B) and cytoplasmic (C) fractions detecting activation/nuclear translocation of NF-κB and TRIM21. Bar graphs represent densitometric analysis of the Western blots with means ± SEMs. *, *P* < 0.05.

It is well established that TRIM21, an E3 ubiquitin ligase, mediates immune signaling and neutralization of antibody-coated viruses via a process of autoubiquitination and deubiquitination (39). Therefore, we next addressed whether recognition of intracellular huMAb-bound ehrlichiae by TRIM21 could also initiate ubiquitination and immune signaling. THP-1 WT and KO cells were incubated with EHRL-4-opsonized *E. chaffeensis*, and samples were examined by confocal microscopy, immunoblot analysis, and quantification of RNA expression for detecting immune signaling and inflammatory response. We detected significant accumulation of K-48- and K-63-linked polyubiquitin (Ub) chains with the intracellular TRIM21-*E. chaffeensis*-EHRL-4 complex using confocal microscopy at 30 min postinfection (Fig. 4A). There was absence of detectable polyUb chains localized with TRIM21 in uninfected cells or in infected cells that were not treated with EHRL-4 (Fig. 4A, top and bottom represent K-63- and K-48-linked Ub chains, respectively). In addition, there was absence of detectable K-63- and K-48-linked polyUb chain recruitment to *Ehrlichia* in both the presence and absence of EHRL-4 antibodies in TRIM21 KO cells, further confirming that ubiquitination of the EHRL-4-opsonized ehrlichia complex involves both antibody-mediated and TRIM21-dependent mechanisms (see Fig. S3A).

**FIG 5:**
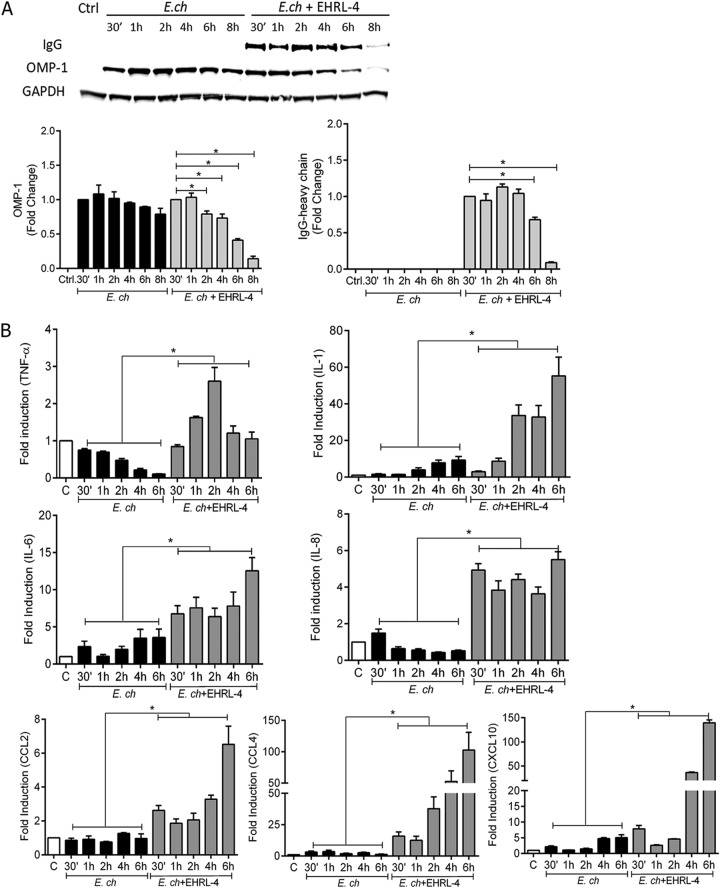
OMP-1 huMAb EHRL-4-*E. chaffeensis* complex detection by TRIM21 mediates degradation and a proinflammatory response. THP-1 cells were infected with *E. chaffeensis* in the presence or absence of EHRL-4, and samples were collected for whole-cell lysates and evaluation of RNA expression at various time points postinfection. (A) Degradation of OMP-1 and EHRL-4 (IgG), with bar graphs representing densitometric analysis of the Western blots with means ± SEMs. *, *P* < 0.05. (B) mRNA expression levels of proinflammatory cytokines and chemokines in THP-1 cells by real-time reverse transcription-PCR. mRNA levels were normalized to GAPDH and compared with the level of uninfected cells. Bar graphs represent means ± SEMs. *, *P* < 0.05.

This accumulation of polyUb chains was accompanied by activation and nuclear translocation of NF-κB and TRIM21 as early as 30 min postinfection (Fig. 4B). In cells with *E. chaffeensis* infection alone, NF-κB nuclear translocation reached a maximum 30 min postinfection and then decreased thereafter. When cells were infected with EHRL-4-opsonized ehrlichiae, NF-κB nuclear translocation was observed at 30 min postinfection and increased for as long as 8 h postinfection (Fig. 4B). Nuclear translocation of TRIM21 was observed only when cells were infected with the EHRL-4-opsonized ehrlichiae. Nuclear translocation of TRIM21 was also observed as early as 30 min postinfection and decreased thereafter (Fig. 4B). Following incubation with EHRL-4-opsonized *E. chaffeensis*, the cytoplasmic levels of NF-κB were increased at 2 to 6 h, and TRIM21 levels decreased at 30 min and 1 h postinfection relative to that with infection alone (Fig. 4C). We also detected OMP-1 and EHRL-4 in the cytosol, and these proteins were largely absent within 6 h postinfection (Fig. 5A). These data suggest that TRIM21 binds intracellular huMAb-coated ehrlichiae, and this binding results in the ubiquitination of the TRIM21-*E. chaffeensis*-EHRL-4 complex and initiation of downstream signaling. Additional analysis of whole-cell lysates of TRIM21 KO cells infected with *Ehrlichia* opsonized with EHRL-4 demonstrated inhibition of OMP-1 and IgG degradation even at 6 h postinfection compared to the significant degradation observed in WT cells (Fig. S3B).

To investigate activation of downstream signaling induced by TRIM21 NF-κB activation, we monitored the expression of cytokine and chemokine mRNA transcripts at early time points by quantitative PCR (qPCR). There was a significant upregulation of proinflammatory cytokine/chemokine transcripts as early as 30 min, which increased for up to 6 h postinfection and remained higher than in cells infected with *E. chaffeensis* alone (Fig. 5B). We observed 3- to 15-fold increases in tumor necrosis factor alpha (TNF-α), interleukin 6 (IL-6), IL-8, and C-C motif chemokine ligand 2 (CCL2) transcripts and 50- to 150-fold increases in IL-1, CCL4, and C-X-C motif chemokine ligand 10 (CXCL10) transcripts in THP-1 cells incubated with EHRL-4-opsonized *E. chaffeensis* (Fig. 5B). IL-12p40 and type 1 interferons, alpha and beta interferons (IFN-α and -β), also exhibited significant increases 30 min postinfection with EHRL-4-opsonized ehrlichiae; however, transcripts decreased thereafter compared to those with *E. chaffeensis* infection alone (see Fig. S4). IFN-β mRNA transcripts increased 2 h postinfection through 6 h in cells infected with EHRL-4-opsonized *E. chaffeensis* (Fig. S4). The TRIM21-mediated proinflammatory response in the presence of EHRL-4-opsonized *E. chaffeensis* was significantly abrogated in TRIM21 KO cells (see Fig. S5). These data demonstrate that binding of huMAb-opsonized *E. chaffeensis* by TRIM21 occurs soon after the complex enters the cell and that this process initiates a rapid proinflammatory response.

### EHRL-4-opsonized *E. chaffeensis* recruited TRIM21 and components of the autophagic apparatus.

**FIG 6:**
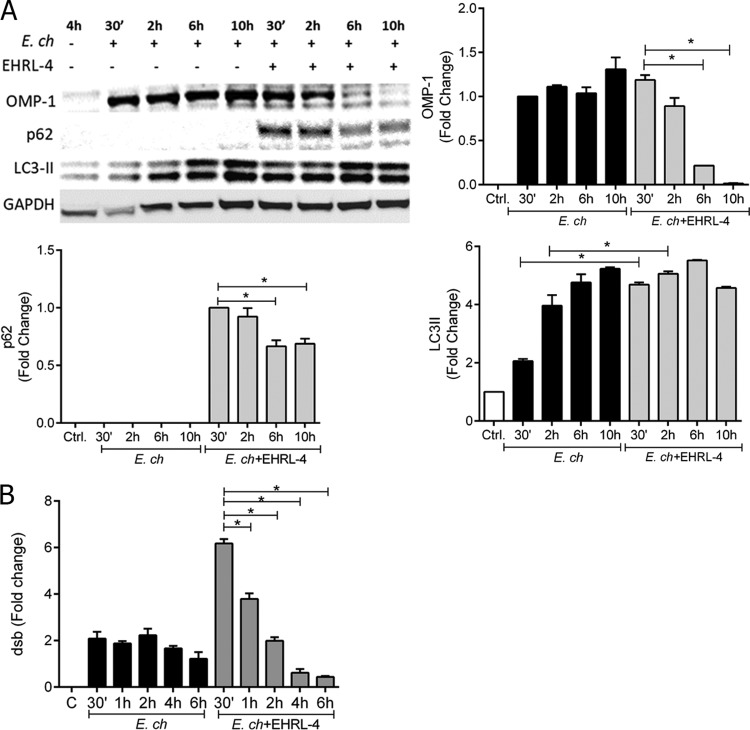
OMP-1 huMAb EHRL-4 mediates intracellular degradation of *E. chaffeensis* by autophagy. THP-1 cells were infected with *E. chaffeensis* in the presence or absence of EHRL-4 and examined for autophagic flux and degradation of ehrlichiae. (A) Western blot analysis of 10 μg of the whole-cell extract showing autophagic flux, detecting accumulation and degradation of p62 and OMP-1 and increasing levels of LC3II when inoculated with EHRL-4-opsonized ehrlichiae. Bar graphs represent densitometric analysis of the Western blots with means ± SEMs. *, *P* < 0.05. (B) Transcript levels of *dsb*, demonstrating bacterial loads at various time points postinfection. Bar graphs represent means ± SEMs. *, *P* < 0.05.

We have demonstrated that EHRL-4-opsonized *E. chaffeensis* enters cells and is immediately recognized by TRIM21; this is followed by a rapid accumulation of polyUb chains, which initiates an inflammatory response concomitant with rapid degradation of ehrlichiae. Because there are two mechanisms for processing ubiquitinated proteins, i.e., the proteasome and autophagy systems, we hypothesized that one of these systems was likely involved in the degradation of opsonized ehrlichiae. In addition, studies have shown that ubiquitinated TRIM21 is a proteasome substrate that assists in the degradation of targeted viral immune complexes (39). This process may also require the participation of unfoldase as well as the segregase enzyme p97/VCP (28). The ehrlichiae are too large to be processed directly by the proteasome system, and TRIM proteins have also been implicated in autophagy (40); so, we first examined the role of autophagy in the degradation EHRL-4-opsonized ehrlichiae by analyzing whole-cell extracts of *E. chaffeensis*-infected cells, and/or EHRL-4-opsonized *E. chaffeensis*, to assess both autophagic flux and ehrlichial degradation. We observed significant degradation of OMP-1 within 6 h postinfection in cells incubated with EHRL-4-opsonized *E. chaffeensis* but not with cells incubated with unopsonized bacteria (Fig. 6A). LC3II, the membrane-associated lipidated form of LC3I, a mammalian homologue of Atg8 and a key indicator of autophagophore formation, was also detected (Fig. 6A) (41). LC3II expression was found to increase during infection, consistent with previous observations that *E. chaffeensis* can initiate autophagy for nutrient acquisition. However, we observed a major increase in LC3II expression early after infection (30 min up to 2 h) with EHRL-4-opsonized *E. chaffeensis*, demonstrating that the host cells rapidly initiate autophagic flux (Fig. 6A). In cells incubated with EHRL-4-opsonized ehrlichiae, there was also a significant accumulation of p62 as early as 30 min, which was followed by significant reduction within the next 6 h, suggesting autophagic degradation. In comparison, there was little accumulation or degradation of p62 in healthy cells and in *E. chaffeensis*-infected cells (Fig. 6A). These LC3II and p62 expression data along with previous studies (42) demonstrate that an autophagic flux occurs early after internalization of EHRL-4-opsonized *E. chaffeensis* (Fig. 6A). Rapid internalization of *E. chaffeensis* was observed as early as 30 min postinfection with the EHRL-4-opsonized ehrlichiae, with the bacterial load decreasing significantly thereafter until 6 h postinfection compared to that with *E. chaffeensis* alone (Fig. 6B). These data reveal that opsonized, but not nonopsonized, ehrlichiae are rapidly internalized and degraded.

**FIG 7:**
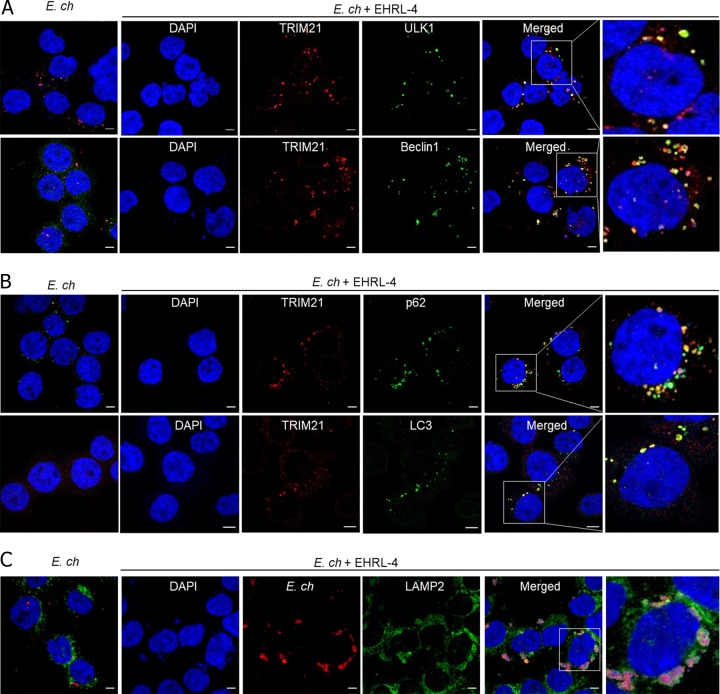
OMP-1 huMAb EHRL-4-opsonized *E. chaffeensis* engages TRIM21, which recruits autophagy regulators and effectors. THP-1 cells were infected with *E. chaffeensis* in the presence or absence of EHRL-4 and examined for interaction with autophagy regulator/effectors at 30 min postinfection. Confocal images showing colocalization of TRIM21 (red) and autophagy regulators ULK1 (green) and beclin 1 (green) (A), colocalization of TRIM21 (red) with sequestosome 1/p62 (green) and mammalian Atg8s (LC3, green) (B), and colocalization of *E. chaffeensis* (red) with LAMP2 (green), only in the presence of EHRL-4 (C). Bars, 10 μm; ×63 magnification (oil immersion lens).

To examine autophagy in the TRIM21-mediated degradation of opsonized ehrlichiae, the distribution and localization of essential autophagy receptors/proteins, as well as TRIM21-IgG-*E. chaffeensis* complexes, were examined by confocal microscopy at 30 min postinfection. Initiation of autophagy requires both Unc-51-like autophagy activating kinase 1 (ULK1) and beclin 1 (43), and both of these factors were localized with cytosolic TRIM21 puncta in cells treated with EHRL-4-opsonized *E. chaffeensis* (Fig. 7A). Endogenous ULK1 was distributed diffusely in *E. chaffeensis*-infected and uninfected control cells and did not colocalize with ehrlichiae in the absence of EHRL-4. There was also significant redistribution and colocalization of the autophagosomal markers LC3 and p62 with puncta in the TRIM21-EHRL-4-*E. chaffeensis*-infected cells, also consistent with the initiation and formation of autophagosomes (TRIMosomes) (Fig. 7B). Although LC3, p62, and beclin 1 were diffusely distributed in uninfected control cells and in *E. chaffeensis*-infected cells, some colocalization of autophagy markers was observed in *E. chaffeensis*-infected cells in the absence of EHRL-4 (see Fig. S6). Previous studies have shown colocalization of LC3, p62, and beclin 1 with the ehrlichial morulae on days 1, 2, and 3 postinfection (44). Hence, the early colocalization we have observed with *E. chaffeensis* in the early stages of infection is consistent with reports of ehrlichia-mediated initiation of autophagosome formation and recruitment to the ehrlichial inclusion for nutrient acquisition. As autophagic degradation requires the autophagosomes to fuse with the lysosomes for degradation of the cargo, we next examined whether the autophagosomes or TRIMosomes undergo lysosomal fusion by examining the distribution and colocalization of lysosome-associated membrane protein 2 (LAMP2) with ehrlichial inclusions (45). Significant colocalization of LAMP2 was observed around the opsonized ehrlichial puncta in cells infected with EHRL-4-opsonized ehrlichiae (Fig. 7C). In contrast, LAMP2 was distributed diffusely in uninfected control and *E. chaffeensis*-infected cells and did not colocalize with ehrlichiae in the absence of EHRL-4 (Fig. S6). These data are consistent with previous reports demonstrating that the ehrlichiae actively evade lysosomal fusion for intracellular survival (44, 46). Collectively, our data suggest that EHRL-4-opsonized ehrlichiae are engaged by TRIM21 and that this factor acts to recruit autophagy receptors and proteins that eventually lead to lysosomal fusion and ehrlichial killing.

### *E. chaffeensis* was degraded intracellularly by TRIM21-mediated selective/precision autophagy.

**FIG 8:**
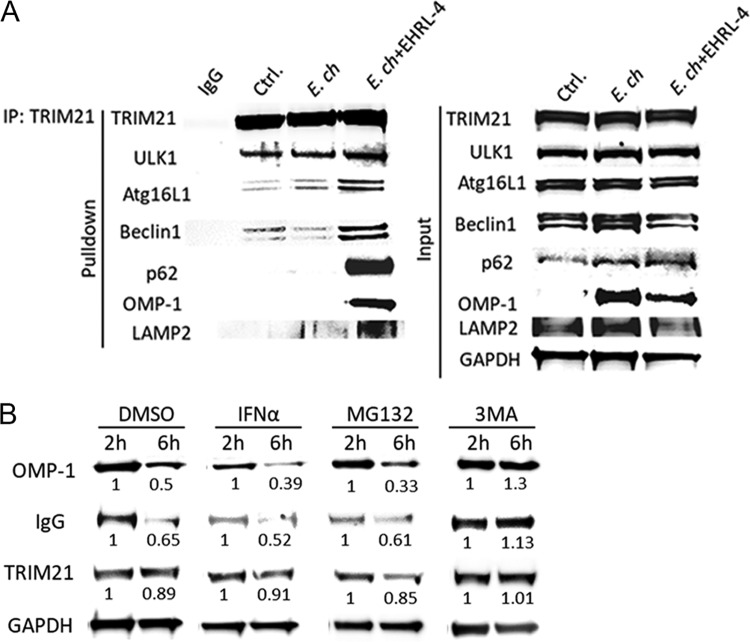
OMP-1 huMAb EHRL-4-mediated degradation of *E. chaffeensis* involves selective autophagy which is abrogated by inhibitor of autophagy. THP-1 cells were infected with *E. chaffeensis* in the presence or absence of EHRL-4 and in the presence or absence of inhibitors of proteasome and autophagy. (A) Western blot analyses of TRIM21 immunoprecipitation demonstrate the interaction of TRIM21 with *E. chaffeensis* OMP-1 and autophagy regulator and effector proteins only in the presence of EHRL-4. Detection of the various regulators, effectors, and *E. chaffeensis* OMP-1 proteins is also shown in 25 μg of the input for Co-IP. (B) Western blot analyses of the whole-cell extracts of THP-1 cells inoculated with EHRL-4-opsonized *E. chaffeensis* and treated with inhibitors of proteasome and autophagy demonstrate the involvement of TRIM21-mediated autophagic degradation of *E. chaffeensis*.

Some TRIM proteins, including TRIM21, can function both as receptors for specific autophagic cargo and as regulators of autophagy, by acting as a platform for the assembly of other autophagy regulators and receptors, thus providing a mechanism for selective autophagy in mammalian cells (31, 47). To confirm that EHRL-4-mediated intracellular autophagic degradation of ehrlichiae was mediated by TRIM21 functioning in this way, we analyzed interactions between TRIM21 and other autophagic regulator and receptor proteins. Using a coimmunoprecipitation (co-IP) assay, we observed that TRIM21 interacted with EHRL-4-opsonized ehrlichiae, because OMP-1 was detected in the TRIM21 complexes (Fig. 8A). TRIM21 also interacted with the key autophagic regulators ULK1 and beclin 1, which are involved in the activation of autophagy. Other TRIM21-interacting factors included autophagy regulators, such as ATG16L1, a component of the autophagy E3-like complex that regulates LC3 conjugation and autophagosome formation, and the autophagic receptor p62. When we examined interactions with proteins associated with autophagic membranes, such as mammalian Atg8 paralogues (LC3), there was no detectable interaction. These data suggest that TRIM21 interacts not only with the IgG-*E. chaffeensis* complex but also with various regulators and effectors of autophagy. Together, these form the basis for selective autophagic degradation of its specific cargo, in this case, *E. chaffeensis*.

Inhibitors of the proteasome (MG132) and the autophagy pathway (3-methyladenine [3-MA]) were used to further address TRIM21-mediated autophagic degradation of EHRL-4-opsonized ehrlichiae. Cells were treated with inhibitors and infected with *E. chaffeensis* or EHRL-4-opsonized *E. chaffeensis*, and OMP-1 and IgG heavy chain degradation was monitored. Treatment with MG132 did not inhibit the degradation of EHRL-4-opsonized *E. chaffeensis* within 6 h postinfection; however, in the presence of 3-MA, the degradation of OMP-1 and IgG was inhibited (Fig. 8B). These data reveal that antibody-mediated degradation of EHRL-4-associated ehrlichiae is dependent on TRIM21-mediated selective autophagy.

## DISCUSSION

Several observations in the past decade support a paradigmatic shift in our understanding of mechanisms of how antibodies can provide protection against intracellular pathogens. Antibodies have been found to be effective in the intracellular environment against both facultative and obligately intracellular bacteria (48, 49). Monoclonal antibodies to the Listeria monocytogenes toxin listeriolysin O (LLO) mediate intracellular neutralization of LLO by a mechanism independent of opsonization and FcR ligation, whereas a human monoclonal antibody to Shiga toxin 2 subunit B intercepts the cell-bound toxin and prevents toxin-mediated cell death intracellularly (50). Antilipopolysaccharide (anti-LPS) antibodies mediate intracellular ADIN of Salmonella enterica by engaging TRIM21 (29). Moreover, antibodies to several viruses such as HIV-1, adenovirus, Sendai virus, influenza, measles, and rotavirus have also been shown to mediate intracellular neutralization (51–54).

These findings have led to the identification of novel mechanisms of antibody-mediated immunity (48, 55). Well-known mechanisms of antibody-mediated protection include opsonization, complement activation, neutralization of toxins and viruses, antibody-dependent cell-mediated cytotoxicity (ADCC), and phagocytosis via extracellular FcγRs (48, 56). Other antibody-mediated immune mechanisms involving nonclassical FcRs include transcytosis of antibodies into intracellular compartments by the neonatal Fc receptor and polymeric Ig receptor, and intracellular neutralization by the high-affinity cytosolic FcR, TRIM21 (57–59).

In recent years, studies with *Ehrlichia* spp. have been important in changing the paradigm regarding the role of antibody-mediated immunity to intracellular bacteria by demonstrating that antibodies play an essential role in protection. Increasing evidence from a number of studies has shown antibodies to *E. chaffeensis* and Ehrlichia muris (subsp. *eauclairensis*) are highly effective in reducing ehrlichial infections even when administered after infection when the bacteria are intracellular (12, 14, 18, 19, 21). Protective humoral immunity to *E. muris* directed against OMP-1 was previously shown in mice to involve classical antibody- and complement-mediated Fcγ receptor-dependent (FcγRI) opsonization mechanisms (24), as reported for most extracellular bacteria (18). In the present study, we have demonstrated the protective efficacy of *E. chaffeensis*-specific huMAbs *in vitro* and determined that intracellular inhibition mechanisms are involved. These findings link the natural protective human immune responses to *E. chaffeensis* with established animal infection models and provide another valuable research tool for studying the mechanisms of antibody-mediated protection.

Three huMAbs were identified that recognize OMP-1, and two of these (EHRL-4 and -15) demonstrated significant inhibition of *E. chaffeensis* infection *in vitro*. The protective huMAbs recognized an immunodominant HVR1 peptide of OMP-1, while the nonprotective OMP-1 huMAb did not. Fine specificity analysis using overlapping peptides within the HVR1 (including the 60 to 90 amino acid residues) indicated that both of the protective huMAb epitopes mapped to similar amino acid sequences within the OMP-1 HVR1. EHRL-15 recognized only the N-terminal peptide (61 to 80 amino acids [aa]) of the HVR1 region, whereas EHRL-4 recognized all the peptides spanning the entire HVR1 region, with higher binding to the nested peptide at 66 to 85 aa. These data indicated the presence of critical interactions in this region, similarly to the epitope of mouse monoclonal antibodies and OMP-1 HVR1 (21). All the huMAbs were of the IgG class, with the protective huMAbs belonging to subclasses IgG3 (EHRL-4) and IgG1 (EHRL-15). The two protective huMAbs demonstrated very strong immunoreactivity to *E. chaffeensis*, whereas the nonneutralizing EHRL-2 was weakly immunoreactive and was not specific to HVR1. These results are consistent with previous studies in mice and humans, where protective *E. chaffeensis* antibodies to OMP-1 that recognized HVR1 had picomolar affinities and long binding half-lives (*t*_1/2_) and belonged to the subclasses IgG2a and IgG3 (12, 14, 21, 60). These observations suggest that epitope recognition within HVR1 of OMP-1, specific isotype and subclass, and affinity are critical criteria contributing to antibody efficacy and mechanism of action against *E. chaffeensis*. We have also demonstrated that linear epitope-specific antibodies to tandem repeat proteins (TRPs) (i.e., TRP32, TRP47, and TRP120, predominantly IgG1) also inhibit *E. chaffeensis* infection *in vitro* and *in vivo* (in immunocompetent and SCID mice) when administered prophylactically or therapeutically. These findings suggest that in addition to the OMP-1 epitopes, TRP epitopes also contribute significantly toward protective human immune responses to *E. chaffeensis* and that protection involves both extracellular and known and novel unknown intracellular mechanisms of action (22).

HuMAbs significantly neutralized *E. chaffeensis* infection *in vitro*, as determined using an *in vitro* neutralization assay. This assay has been used to examine prophylactic and therapeutic efficacy of anti-TRP antibodies against *E. chaffeensis* infection (22) and provides a model to monitor inhibition pre- and postehrlichial internalization. We have demonstrated that infection in the presence of EHRL-15 prevented ehrlichial internalization, suggesting that this HuMAb acts in extracellular neutralization or blocking of ehrlichial attachment. Infection in the presence of EHRL-4, in contrast, mediated entry of a large number of opsonized ehrlichiae, and these colocalized with TRIM21. These observations demonstrate that treatment with neutralizing OMP-1-specific huMAbs that exhibit subtle differences in HVR1 epitope reactivity and are composed of distinct IgG subclasses (IgG1 and IgG3) results in antibody-mediated protection against *E. chaffeensis* via distinct mechanisms. Moreover, TRIM21 ablation reversed the neutralizing abilities of EHRL-4 without affecting the neutralization mediated by EHRL-15, further confirming that different mechanisms of neutralization are employed by these different OMP-1-specific huMAbs.

Functional diversity of IgGs is influenced by IgG subclasses, which in turn affects binding to different Fc receptors (61). Both IgG1 and IgG3 bind to all human FcγRs, with IgG3 having higher affinity for FcγRIII, a low-affinity FcγR present on natural killer (NK) cells, monocytes/macrophages, and dendritic cells (62). IgG1 can elicit extracellular responses such as antibody-dependent cell-mediated cytotoxicity (ADCC), antibody-dependent cellular phagocytosis (ADCP), and complement-dependent cytotoxicity (CDC). Human IgG3 can activate complement- and FcγR-mediated functions more effectively than other subclasses but has a short half-life compared to those of the other IgGs (63). Both IgG1 and IgG3 can bind to the IgG-salvage receptor (FcRn) and could in principle be subjected to FcRn-mediated transcytosis, which may also mediate intracellular neutralization of pathogens. FcRn-mediated transcytosis is efficient for both IgG1 and IgG3 and reduces catabolism, thereby enhancing antibody half-life (64). FcRn is also expressed in macrophages and dendritic cells, facilitating transport of IgG-bound antigens through intracellular routes to favor antigen presentation and subsequent immune responses. Therefore, a combination of these differences in IgG1 and IgG3 subclasses and their interactions with the various FcRs along with their ability to block ehrlichial binding and entry could account for the different mechanisms of action employed by these huMAbs. Antibodies that fail to block entry, or do not engage antibody-mediated effector functions in the extracellular environment, may enter the cells and engage TRIM21, which has broad specificity for all isotypes and subclasses (57, 65).

The present study is the first report of TRIM21 involvement in antibody-mediated immune responses and degradation of an obligate intracellular bacterium. EHRL-4-opsonized ehrlichiae were internalized, leading to TRIM21 engagement and ehrlichial killing. TRIM21 is a high-affinity cytosolic IgG receptor that belongs to a group of E3 ligases implicated in the regulation of a variety of cellular functions, including cell cycle progression, autophagy, innate immunity, and antiviral responses (25, 40, 66–68). TRIM proteins are characterized by their conserved RBCC domain, which includes a novel (really interesting new gene [RING]) E3 ligase domain (R), one or two B-box domains (B), and a coiled-coil domain (CC) (69). The variable C-terminal regions of TRIMs are responsible primarily for their interactions with target proteins, and TRIM21 binds the Fc portion of IgG at residues overlapping the CH2 and CH3 domains via its carboxyl-terminal B30.2 (PRYSPRY) (70). TRIMs have been implicated in regulating responses to viral infection and are known to exert their effector functions, such as assembly of signaling complexes, proteolytic degradation, initiation of autophagy, subcellular localization, and host transcription, by mediating ubiquitination of target proteins (66, 71). TRIM21 is also implicated in innate immune responses directed against parasitophorous vacuole (PV)-resident pathogens such as *Toxoplasma* and *Chlamydia*. PV destruction is dependent on decoration of PVs with ubiquitin, mediated by E3 ubiquitin ligases TRAF6 and TRIM21, eliciting p62-mediated escort and deposition of guanylate binding protein (GBPs), thereby resulting in the elimination of PVs and their microbial inhabitants (72, 73).

Recognition of the EHRL-4-*E. chaffeensis* complexes by TRIM21 resulted in a rapid accumulation of K-48 and K-63 mediated polyubiquitin chains, a concurrent nuclear translocation of NF-κB and TRIM21, an innate immune response associated with increased expression of proinflammatory cytokine and chemokine genes, and simultaneous degradation of OMP-1 and IgG. These observations are similar to those from studies implicating TRIM21-Fc interactions in the antibody-dependent intracellular neutralization (ADIN) of nonenveloped viruses and for facultative intracellular bacteria such as Salmonella enterica (27, 29). ADIN was substantially reduced following genetic deletion of TRIM21 or after site-directed mutagenesis of the TRIM21 binding site on the neutralizing antibody (74). These observations are attributed to TRIM21’s dual effector and sensor roles, as it not only mediates ADIN but also initiates an innate immune response upon infection with antibody-coated pathogens by recruiting the proteasomal system for degradation and simultaneously activate transcription factors (NF-κB/IRFs/AP-1) resulting in a proinflammatory response (27, 28, 75). TRIM21-mediated dual responses are coordinated by sequential autoubiquitination by K63, followed by K48 polyUb linkages, recruiting the proteasome resulting in pathogen degradation, and liberation of K63-linked Ub chains by deubiquitination by the proteasome-associated deubiquitinase Pho1, thereby stimulating immune signaling (39). TRIM21 can also directly induce the formation of unanchored K63 polyUb chains, inducing transforming growth factor beta-activated kinase 1 (TAK1)-dependent NF-κB activation (27). TRIM21-mediated release of the degradation products, such as viral genomes, can also act as a precursor for a second wave of immune signaling (76).

TRIM21 is predominantly located in the cytoplasm but translocates to the nucleus under certain conditions, such as proinflammatory stimulation (77, 78). We observed nuclear translocation of NF-κB, as well as TRIM21 and p62, in response to treatment with EHRL-4-opsonized *E. chaffeensis*. In addition, there was an initial spike in IL-12p40 and type 1 IFN followed by a gradual decrease over time. A recent study demonstrated that TRIM21-mediated intracellular antibody signaling is regulated by the B-Box, which represses TRIM21 ubiquitination and immune activation in the absence of infection. Upon infection, TRIM21 is derepressed by IKKβ and TANK-binding kinase 1 (TBK1)-mediated phosphorylation in the RING domain, promoting E2 binding, RING catalysis, NF-κB activation, and cytokine transcription (79). In the nucleus of activated macrophages, TRIM21 interacts with p62 and IRF8, wherein p62 is suggested to play a critical role in regulating the TRIM21-IRF8 interactions via ubiquitination, thereby regulating IRF-8 and NF-κB activation of cytokine and autophagy genes (80–83). Other reported substrates for TRIM21-mediated ubiquitination include IRF3, IRF5, and IRF7, which affects expression of IRF target genes (IFN-β, IL-6, and IL-12p40). This in turn tightly regulates the expression of TRIM21 as a negative feedback loop and is essential in regulating TRIM21 immune responses (84–86). Hence, TRIM21 has specific cytoplasmic (UBE2D1) and nuclear (UBE2E1) E2s, and translocation to the nucleus under proinflammatory conditions clearly shows that TRIM21 has both cytoplasmic and nuclear substrates, which are regulated via ubiquitination (87).

Ours is the first confirmed report of autophagic degradation of an intracellular pathogen, *E. chaffeensis*, by TRIM21-mediated selective/precision autophagy. Treatment of cells with opsonized *E. chaffeensis* resulted in a rapid degradation of the ehrlichia-huMAb complex that coincided with autophagic flux (i.e., increased LC3II levels and accumulation and degradation of p62). Abrogation of ehrlichial degradation in the presence of an autophagy inhibitor and the detection of various autophagy regulators and effectors interacting with TRIM21 and opsonized ehrlichiae confirmed the involvement of autophagy in the TRIM21-mediated ADIN of opsonized *E. chaffeensis*. Autophagy is a highly conserved and tightly regulated major intracellular degradation system in eukaryotic cells in addition to the ubiquitin-proteasome system. Several studies have linked TRIM proteins to autophagy, showing that TRIMs can act both as receptors identifying specific cargo and as regulators of autophagy. TRIMs are known to act as a platform (TRIMosome) for the assembly of the key autophagic regulators and effector proteins (ULK1, beclin 1, mammalian Atg8s [mAtg8s], and p62) to form autophagosomes mediating selective autophagic degradation of highly specific targets (47, 88). As many as 24 different TRIM proteins are required for induction of IFN-γ-induced autophagy (89). TRIM proteins interact with components of the autophagic apparatus, including activated ULK1 and beclin 1, and most TRIMs that regulate autophagy can also interact with both LC3 and p62 (47). TRIM21 specifically binds the activated dimeric form of IRF3 via its C-terminal SPRY domain and interacts with ULK1, beclin 1, mAtg8s, and p62, leading to autophagic degradation of activated IRF3, dampening the type 1 IFN responses. This process was termed “precision autophagy,” as TRIM21 acts as autophagic receptor regulator executing precision autophagy of specific cytoplasmic targets by direct target recognition via specific protein-protein interaction (30, 31). Another study demonstrated that TRIM21-mediated monoubiquitination of active IKKβ routes it to the autophagosomes for degradation, thereby downregulating IKKβ-induced NF-κB signaling. A study of TRIM21-mediated ADIN of opsonized *Salmonella* also detected TRIM21 and *Salmonella* colocalizing with LC3, suggesting that involvement of autophagy was likely, but this was not further explored or confirmed (29).

**FIG 9:**
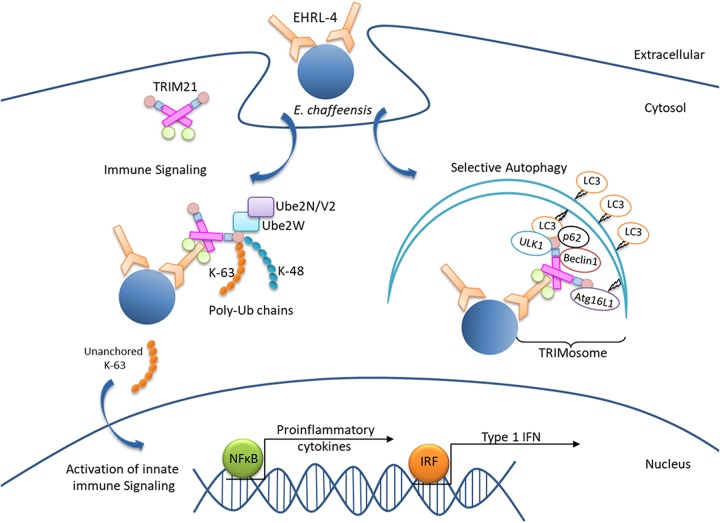
Proposed model of OMP-1 huMAb EHRL-4-dependent TRIM21-mediated immune signaling and degradation of *E. chaffeensis* by a process of TRIM21-mediated selective autophagy.

Our study demonstrates that humoral immunity in humans is effective against the intracellular pathogen *E. chaffeensis* and shows that protective antibodies differing in antigenic epitope recognition, isotype, subclass, and affinity employ different mechanisms of action targeting the pathogen in both the extracellular and intracellular compartments. This study also demonstrates that a unique combination of TRIM21-mediated rapid intracellular sensing induces an immune response to opsonized *E. chaffeensis* by directly binding it and by simultaneously recruiting autophagic machinery, thus coordinating target recognition and initiation of autophagy for selective degradation of the pathogen. A model for this pathway of intracellular killing is summarized in Fig. 9. Our data also demonstrate that anti-*E. chaffeensis* huMAbs are potential candidates for immunotherapy.

## MATERIALS AND METHODS

### Cell culture and *E. chaffeensis*.

Human monocytic leukemia cells (THP-1; ATCC TIB-202) were propagated in RPMI 1640 with l-glutamine and 25 mM HEPES buffer (Invitrogen), 1 mM sodium pyruvate (Sigma, St. Louis, MO), 2.5 g/liter d-(+)-glucose (Sigma), and 10% fetal bovine serum at 37°C in a 5% CO_2_ atmosphere. *E. chaffeensis* (Arkansas strain) was propagated in THP-1 cells as previously described (90). For antibody inhibition experiments, host cell-free *E. chaffeensis* was prepared by rupturing infected THP-1 cells using 1-mm-diameter sterile glass beads and vortexing. Briefly, infected THP-1 cells were harvested and pelleted by light centrifugation (500 × *g* for 15 min). The pellet was resuspended in sterile phosphate-buffered saline (PBS) in a 50-ml tube containing glass beads and vortexed. The cell debris was pelleted (1,500 × *g* for 10 min), and the supernatant was further pelleted by high-speed centrifugation (10,000 × *g* for 15 min, 4°C). The purified ehrlichiae were resuspended in PBS, and whole *E. chaffeensis* antigen for ELISA and Western blotting was prepared as described previously (91). Briefly, infected THP-1 cells were harvested (500 × *g*, 15 min), resuspended in PBS, and sonicated (40 Hz) twice for 10 s, and cell debris was removed by low-speed centrifugation (1,500 × *g*, 10 min, 4°C). The cell-free ehrlichiae in the supernatant were pelleted (10,000 × *g*, 15 min, 4°C) and washed in PBS, the protein concentration was determined, and the lysate was frozen at −80°C.

### PBMC isolation and hybridoma generation.

The study was approved by the Vanderbilt University Medical Center Institutional Review Board. Peripheral blood was collected from healthy donors with history of HME infection after written informed consent. Peripheral blood mononuclear cells (PBMCs) from the donors were isolated by density gradient separation on Ficoll, cryopreserved, and stored in the vapor phase of liquid nitrogen until use. Human hybridoma cell lines secreting human MAbs were generated as described previously (92). Briefly, human B cells in the PBMC suspension were immortalized by transformation with Epstein-Barr virus (EBV) in the presence of CpG10103, cyclosporine, and a Chk2 inhibitor and plated in 384-well culture plates. On day 8, the supernatants from transformed B cells were used to screen for the presence of antibodies that bound to *E. chaffeensis* antigens by ELISA. Cells from the wells containing B cells secreting antigen-reactive antibodies were fused with HMMA2.5 myeloma cells using a BTX ECM 2001 electro cell manipulator. After fusion, human hybridomas were selected in medium with hypoxanthine-aminopterin-thymidine (HAT) solution containing ouabain. The hybridomas were cloned by flow cytometric sorting of single cells into 384-well plates and then expanded in culture. Particular clones for downstream studies were selected by choosing the clone for each independently derived hybridoma line that exhibited the highest level of IgG secretion.

### Production of IgG for *E. chaffeensis*-specific MAbs from hybridoma cells.

The selected cloned cell lines secreting *E. chaffeensis*-specific MAbs were grown initially in hybridoma growth medium (ClonaCell-HY medium E, 03805; STEMCELL Technologies) and then switched to serum-free medium (GIBCO Hybridoma-SFM, 12045084; Invitrogen) for antibody expression and purification. IgGs from the hybridoma cell line supernatants were purified by affinity chromatography using protein G columns (Protein G HP columns; GE Life Sciences). Purified IgG generated from hybridomas was used for all studies, including ELISA, Western blotting, and immunofluorescence assay (IFA) for reactivity to whole *E. chaffeensis* and recombinant immunoreactive proteins.

### Neutralization assay and quantification of *E. chaffeensis*.

A neutralization assay, consisting of an *in vitro* cell-based assay to test the antibody-mediated protection against *Ehrlichia* infection, was used to determine the protection efficacy of the huMAbs. This assay was previously developed in our laboratory to evaluate the ability of *E. chaffeensis* epitope-specific antibodies to inhibit infection (22). Briefly, THP-1 cells (10^5^ cells/well) were plated in 96-well round-bottom plates in serum-free medium, treated with 5 μg/ml of huMAbs or control IgG diluted in PBS for 2 h, and then incubated with cell-free *E. chaffeensis* at a multiplicity of infection (MOI) of 50. Samples were collected at various intervals postinfection, and the absolute *E. chaffeensis dsb* copy number was determined by real-time qPCR and plotted against the standard curve, as previously described (93). THP-1 cells were washed with PBS and lysed in SideStep lysis and stabilization buffer (Agilent Technologies, Santa Clara, CA), and real-time quantitative PCR (qPCR) amplification was performed using Brilliant II SYBR green Mastermix (Agilent), forward primer (5′-GCTGCTCCACCAATAAATGTATCCCT-3′), and reverse primer (5′-GTTTCATTAGCCAAGAATTCCGACACT-3′), using an EP Realplex2 S Mastercycler (Eppendorf). The absolute *E. chaffeensis dsb* copy number in the cells was determined against an external standard curve, or the fold change of *dsb* copy number relative to the control was normalized to qPCR-detected levels of the host genomic glyceraldehyde-3-phosphate dehydrogenase (*gapdh*) gene.

### Reverse transcription-PCR quantification.

Total RNA was extracted from THP-1 cells, which were uninfected or infected with or without huMAb treatment, using an RNeasy Mini kit (Qiagen) according to the manufacturer’s instructions, as described previously (94). Briefly, an RNase-free DNase set (Qiagen) was used for on-column DNA digestion, and cDNA was synthesized from total RNA (1 μg) using an iScript cDNA synthesis kit (Bio-Rad). The expression levels of proinflammatory cytokines and chemokines and interferon-stimulated genes were quantitated by qPCR using Brilliant II SYBR green qPCR master mix (Agilent Technologies) and gene-specific primers (Integrated DNA Technologies, IL, USA). Gene expression values were normalized to GAPDH expression and calculated using the threshold cycle (2^−ΔΔ^*^CT^*) method.

### Reagents, antibodies, and inhibitors.

*E. chaffeensis*-specific huMAbs used in this study were provided by the Vanderbilt Immunology Core as described above. Other antibodies used in this study for immunofluorescence microscopy, immunoblot, and immunoprecipitation assays include polyclonal rabbit anti-dsb antibody (95), anti-TRIM21 (sc-25351, sc-20960), LC3α/β (H-47; sc-292354), anti-p62/SQSTM1 (H-290; sc-25575), anti-BECN1 (E-8; sc-48341), and ULK1 (F-4; sc-390904) from Santa Cruz Biotechnology (Dallas, TX); anti-LAMP-2 (H4B4) from Developmental Studies Hybridoma Bank (University of Iowa); anti-GAPDH (10494-1-AP) from Proteintech (Rosemont, IL); and anti-K63-linkage-specific polyubiquitin (D7A11; 5621S), anti-K48-linkage-specific polyubiquitin (D9D5; 12805S), anti-NF-κB (D14E12; 8242S), anti-p62/SQSTM1 (D5E2; 8025S), anti-Atg16L1 (D6D5; 8089S), anti-ULK1 (D8H5; 8054S), and anti-LC3A/B (D3U4C, 12741S) from Cell Signaling Technology (Danvers, MA). Pharmacological inhibitors of the proteasome and autophagy pathways used in this study include MG-132 (474791; Calbiochem, La Jolla, CA) and 3-methyladenine (3-MA; M9281-100MG; Sigma, St. Louis, MO). The synthetic OMP-1 HVR-1 peptides (GenScript, Piscataway, NJ) used in ELISAs include TTVGVFGLKQNWDGSAISNSSPNDVFTVSN (aa 60 to 90), TTVGVFGLKQNWDGSAISNS (aa 61 to 80), NWDGSAISNSSPNDVFTVSN (aa 71 to 90), and FGLKQNWDGSAISNSSPNDV (aa 66 to 85).

### ELISA and antibody isotyping.

ELISA was used to determine the reactivity and fine specificity of the huMAbs to the OMP-1 HVR1 synthetic peptides. The synthetic peptides were supplied as a lyophilized powder and resuspended (1 mg/ml) in phosphate-buffered saline (PBS). MaxiSorp ELISA plates (Nunc, Roskilde, Denmark) were coated with 1.0 μg/well of the respective synthetic peptides in PBS (pH 7.4), and the assay was performed as previously described with modifications (33). Briefly, after overnight coating at 4°C, plates were washed thrice with 200 μl PBS with Tween 20 (0.2%) (PBST), blocked with 2% milk diluted in Starting Block (PBS) blocking buffer (37538; Thermo Scientific) at room temperature (RT) for 1 h, and washed again with PBST. HuMAbs were diluted (1:1,000) in blocking buffer and added to the wells for 1 h at RT with shaking. The ELISA plates were then washed and incubated with 1:5,000 diluted peroxidase-labeled goat anti-human IgG (H+L) (Kirkegaard & Perry Laboratories) for 1 h at RT with shaking. The optical density of the samples at 650 nm (OD_650_) was measured using a microplate reader (VersaMax; Molecular Devices, Sunnyvale, CA), and data were analyzed using SoftMax Pro v4.0 (Molecular Devices). OD_650_ readings represent the means from three wells (± standard deviations). IgG subclass identification of the huMAbs was determined using the Human IgG Subclass Profile kit from Invitrogen (number 991000), according to the manufacturer’s instructions.

### Immunofluorescence assay and confocal microscopy.

The reactivity of huMAbs to *E. chaffeensis* was determined by IFA as described previously (96). Antigen slides were prepared from DH82 cells infected with *E. chaffeensis* (Arkansas strain), which were applied to each well of 12-well slides, air dried, and acetone fixed. HuMAbs were diluted 5-fold in PBS (1:100 to 1:12,500), and 10 μl of each dilution was added to each well. Slides were incubated at 37°C for 30 min, washed twice in PBS, and air dried. The huMAbs were detected using Alexa Fluor 488-conjugated goat anti-human IgG (H+L) secondary antibody (1:100, A-11013; Invitrogen).

*E. chaffeensis*-infected THP-1 cells in the presence or absence of huMAbs were collected at different time points postinfection and adhered to glass slides by cytocentrifugation. Cells were fixed with 4% paraformaldehyde (PFA) for 15 min at RT followed by two washes with PBS. Slides were blocked and permeabilized with 0.3% Triton X-100 in 2% bovine serum albumin (BSA) in PBS for 30 min, washed with PBS, and incubated with primary antibody (1:100) diluted in PBS with 2% BSA for 1 h. After washing, slides were incubated with Alexa Fluor 568 IgG (H+L) and/or Alexa Fluor 488 IgG (H+L) secondary antibodies (1:100; Invitrogen) for 30 min, washed, and mounted with ProLong Gold Antifade reagent with DAPI (4′,6-diamidino-2-phenylindole) (Invitrogen). Immunofluorescence images were captured with an Olympus BX61 epifluorescence microscope and analyzed using Slidebook software (ver. 5.0; Intelligent Imaging Innovations, Denver, CO) (94). Confocal laser micrographs were obtained with Zeiss LSM 880 laser microscope and analyzed with Zen black software (97).

### Generation of TRIM21 knockout cells using CRISPR-Cas9 genome editing.

CRISPR-Cas9 was used to knockout (KO) TRIM21 in THP-1 cells, as described previously with minor modifications (98). Single guide RNAs (sgRNAs) for the TRIM21 gene were designed using the ChopChop Tool (99) in two different exons and cloned into the Esp3I restriction site of lentiviral CRISPR plasmid (lentiCRISPR ver. 2.0, plasmid number 52961; Addgene) (100), and the clone was confirmed by sequencing. For lentivirus production, equal amounts of vesicular stomatitis virus G (VSV-G), psPAX2 (plasmid number 12260; Addgene), and LentiCRISPRv2 (containing the guide RNA of interest) were transfected into HEK293T cells with MIR 2704 (TransIT-293 reagent, Mirus Bio, WI, USA). The viral supernatant was harvested at 48 h posttransfection, filtered through 0.45-μm filters (Millipore), and applied to THP-1 cells. After transduction (48 h), 2 μg/ml of puromycin was added to the cells, and following selection, cells were collected for KO assessment by Western blotting and phenotypic analysis. The nontarget control cells transfected with the empty plasmid were used as a reference control.

### Western immunoblot analysis.

THP-1 cells were washed with ice-cold PBS and subjected to either whole-cell lysate preparation or nuclear and cytoplasmic fractionation. Whole-cell lysates were prepared as previously described (94), and cytoplasmic and nuclear extracts were prepared using the NE-PER kit (number 78833; Thermo Fisher Scientific) according to the manufacturer’s instructions. Protein concentrations of lysates and extracts were determined using the Pierce bicinchoninic acid (BCA) protein assay kit (number 23225; Thermo Fisher Scientific) and then stored at –80°C. Equal amounts of protein (10 to 25 μg/well) were separated by sodium dodecyl sulfate-polyacrylamide gel electrophoresis (SDS-PAGE), transferred to a nitrocellulose membrane, and immunoblotted with primary antibodies. Horseradish peroxidase-conjugated anti-rabbit or anti-human IgG (H+L) secondary antibodies (Kirkegaard & Perry Laboratories, Gaithersburg, MD) were used for detection, and the immunoblots were visualized using SuperSignal West Dura chemiluminescent substrate or enhanced chemiluminescence (ECL) (Thermo Fisher Scientific) in a ChemiDoc-It2 515 imager (UVP, Upland, CA).

### *In vitro* huMAb opsonization and pharmacological inhibitor studies.

THP-1 cells (10^6^ cells/ml) were seeded in triplicates/condition in 6-well cell culture plates (Corning Inc., Kennebunk, ME) in serum-free medium. Cells were incubated with medium or with cell-free *E. chaffeensis* (MOI of 50) with or without opsonization with huMAbs. For opsonization, cell-free *E. chaffeensis* was incubated with the huMAbs for 15 min at 37°C before incubation with cells. For treatment with pharmacological inhibitors (proteasome or autophagy), cells were pretreated with the inhibitors (25 μM MG132 or 10 mM 3-MA) or dimethyl sulfoxide (DMSO) for 2 h before infection. Samples were collected at various time points postinfection for IFA, immunoblots, and qPCR analysis to determine the roles of proteasome and autophagy in *E. chaffeensis* infection, recruitment of various autophagic regulators/receptors, and degradation of *E. chaffeensis*. Cell viability was assessed by trypan blue dye exclusion test and Diff-Quick staining (101).

### Coimmunoprecipitation.

Coimmunoprecipitation (Co-IP) was performed using the Crosslink Immunoprecipitation kit (Pierce) as described previously (102) with modifications. Briefly, THP-1 cells (10^7^) were infected with host cell-free *E. chaffeensis* incubated with or without huMAbs and uninfected cell controls. Cells were harvested 30 min postinfection, washed with ice-cold PBS, resuspended in 1 ml of ice-cold IP lysis buffer (Pierce) containing protease inhibitor cocktail (Sigma), phosphatase inhibitor cocktail (Thermo Scientific), phenylmethylsulfonyl fluoride, and *N*-ethylmaleimide, and then incubated on ice for 20 min with mild vortexing. Cell lysates (750 μg) were incubated with protein A/G magnetic beads (Pierce) and cross-linked with an anti-TRIM21 mouse monoclonal antibody or isotype control for 1 h at RT. The beads were washed with IP lysis buffer, and the bound antigen was eluted, solubilized in SDS sample loading buffer, separated on a 4% to 20% Bis-Tris gel (GenScript), and transferred to a nitrocellulose membrane. The membrane was probed with TRIM21 antibody to confirm the pulldown of TRIM21 and then probed for the presence of OMP-1 and various regulators and effectors of autophagy.

### Statistical analysis.

Data were obtained from at least three independent biological replicates performed in triplicates, and results are expressed as means ± standard deviations (SDs), unless otherwise indicated. Differences between means from experimental groups were evaluated using two-tailed Student’s *t* tests (Prism 6; GraphPad Software, La Jolla, CA). A *P* value of <0.05 was considered statistically significant.

## Supplementary Material

Supplemental file 1
