# Supplementary material for: Ehrlichia chaffeensis Outer Membrane Protein 1-Specific Human Antibody-Mediated Immunity Is Defined by Intracellular TRIM21-Dependent Innate Immune Activation and Extracellular Neutralization
Source: Infect Immun. 2019 Nov 18;87(12):e00383-19. doi: 10.1128/IAI.00383-19 (PMC6867850; doi:10.1128/IAI.00383-19)
Supplement: Supplemental file 1 [file IAI.00383-19-s0001.pdf]

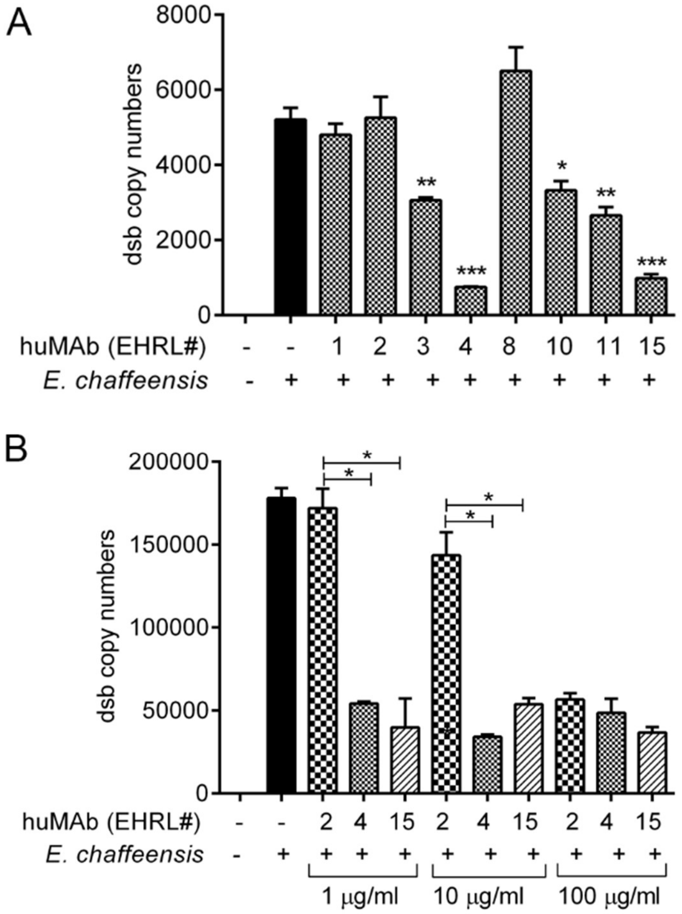

Fig. S1. *In vitro* protection by *E. chaffeensis*-specific huMAbs. (A) Eight different *E. chaffeensis* huMAbs were tested for their ability to inhibit ehrlichial infection *in vitro*. THP-1 cells were preincubated with antibodies then infected with ehrlichiae and infection status determined on day 3 post infection by qPCR. **(B)** To determine the optimal neutralizing activity of huMAbs EHRL-2, 4 & 15, different concentrations the antibodies (1 µg to 100 µg/mL) were tested by *in vitro* ehrlichial neutralization assay and infection status determined on day 3 post infection by qPCR. Bar graphs represent means ± SEM, (*p <0.05).

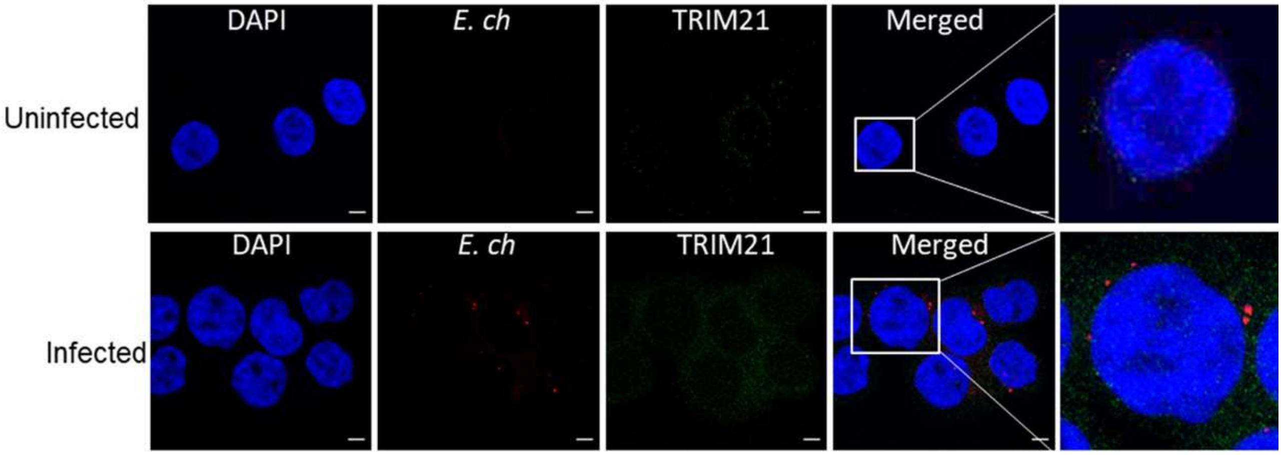

Fig. S2. *E. chaffeensis* infection and interaction with TRIM21. Uninfected and *E. chaffeensis*-infected THP-1 cells were fixed 30 min post infection and analyzed by immunofluorescent confocal microscopy. Confocal images show internalization of *E. chaffeensis* (red) and the diffusely distributed TRIM21 (green). Cells were visualized under 63x oil immersion lens and the scale bar represents 10 μm.

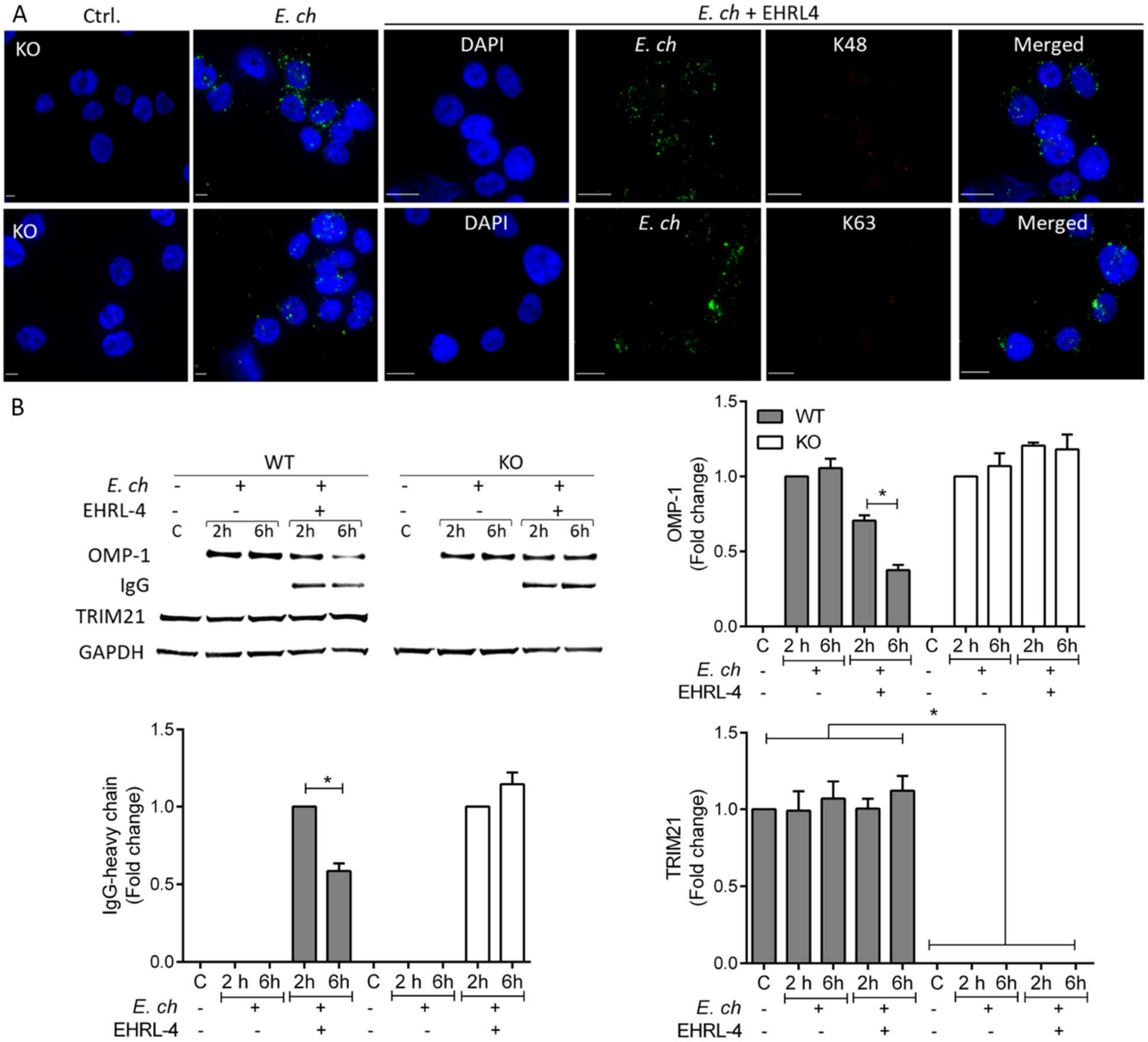

Fig. S3. Analysis of ubiquitination and degradation of EHRL-4-*E. chaffeensis* complexes in TRIM21 KO cells. TRIM21 KO were infected with *E. chaffeensis* in the presence or absence of EHRL-4. Samples were collected 30 min post infection for immunofluorescence microscopy and whole cell extracts were prepared at 2 and 6 h post infection for Western blot analysis. **(A)** Immunofluorescence images show ehrlichiae in green and the recruitment of K63 and K48 linked polyUb chains in red; Scale bar = 10 μm, magnification = 63x oil immersion. The Ctrl. and *E. ch* panels represent the merged images for *E. chaffeensis* and K63/48 linked Ub chains in uninfected and infected KO cells. **(B)** Western blot analysis of whole cell lysates demonstrates

the inhibition of degradation of the OMP-1 and IgG heavy chain in the absence of TRIM21 as compared to the significant degradation observed in WT cells, post infection in the presence of EHRL-4. Bar graphs represent densitometric analysis of the Western blots (mean ± SEM and *$p<0.05$).

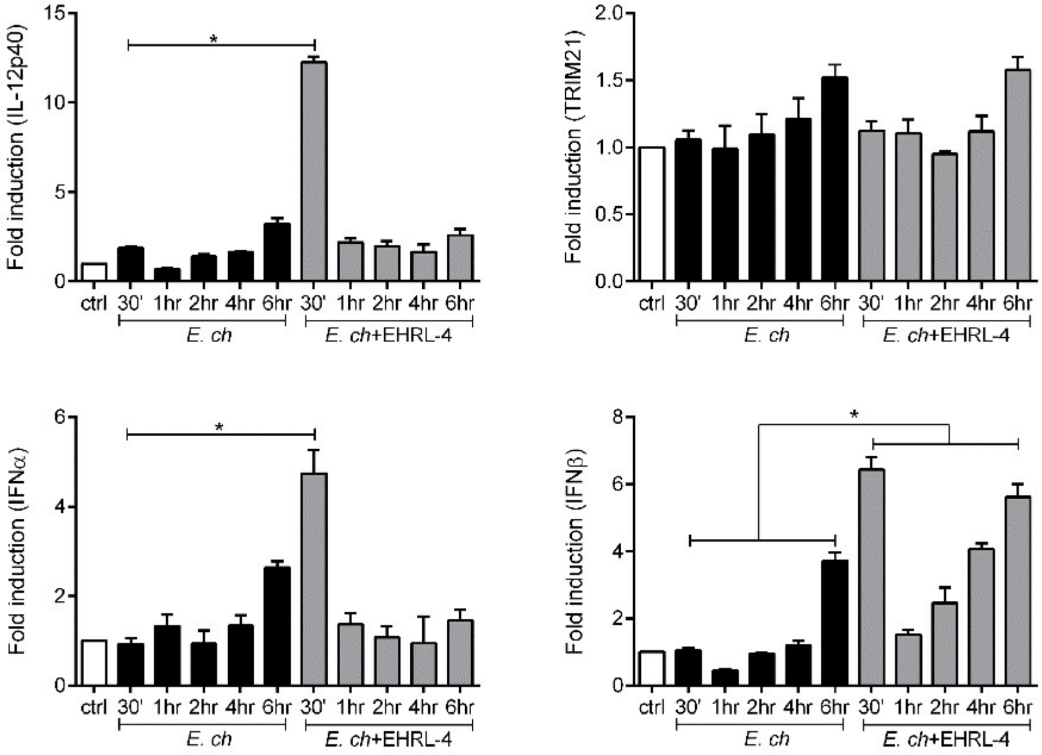

Fig. S4. EHRL-4-opsonized *E. chaffeensis* induction of type 1 IFNs and IL-12p40. THP-1 cells were incubated with *E. chaffeensis* in the presence or absence of EHRL-4, and samples were collected for RNA isolation at various time points post infection. Expression levels of type-1 IFNs, IL-12p40 and TRIM21 were analyzed using real-time RT-PCR. mRNA levels were normalized to GAPDH and compared with the level of uninfected cells. Bar graphs represent means ± SEM; *p<0.05.

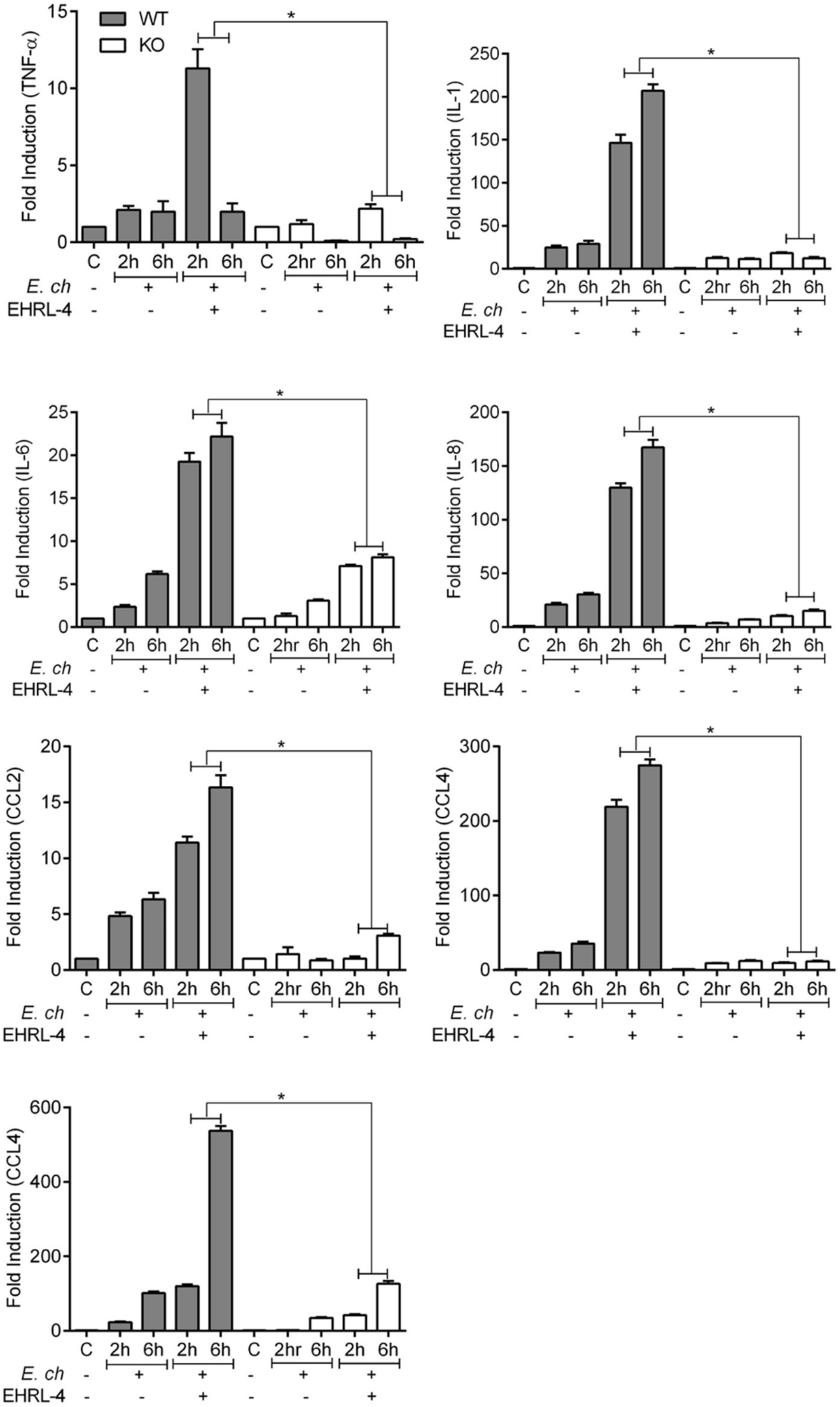

Fig. S5. Analysis of proinflammatory response initiated by EHRL-4-*E. chaffeensis* complexes in TRIM21 KO cells. THP-1 WT and TRIM21 KO cells were infected with *E.*

chaffeensis in the presence or absence of EHRL-4, and samples were collected for evaluation of mRNA expression levels of proinflammatory cytokines and chemokines at 2 and 6 h post infection by real-time RT-PCR. mRNA levels were normalized to GAPDH and expression levels in KO cells were compared with the levels in WT cells. Bar graphs represent means ± SEM; *$p<0.05$.

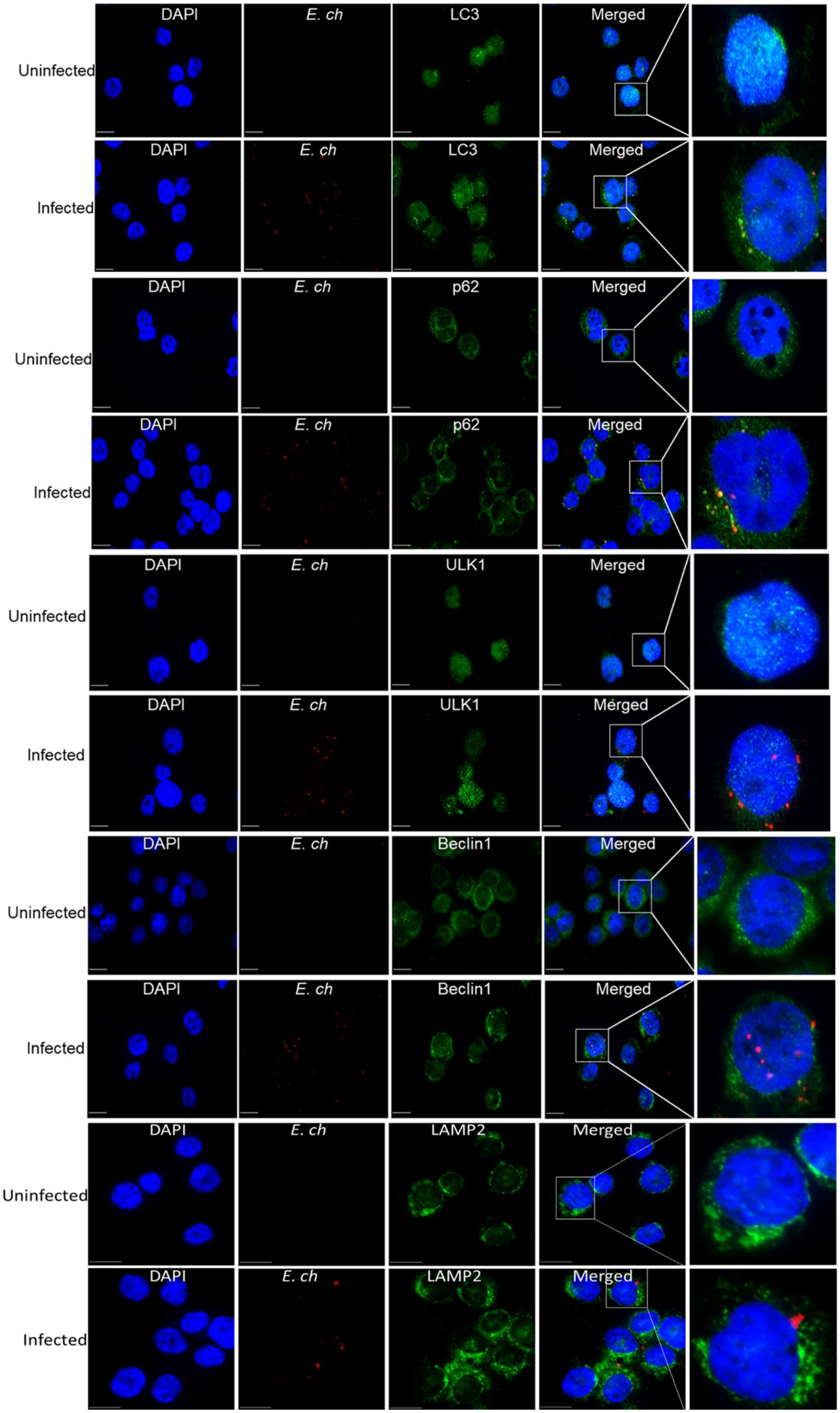

DAPI
E. ch
LC3
Merged
Uninfected
DAPI
E. ch
LC3
Merged
Infected
DAPI
E. ch
p62
Merged
Uninfected
DAPI
E. ch
p62
Merged
Infected
DAPI
E. ch
ULK1
Merged
Uninfected
DAPI
E. ch
ULK1
Merged
Infected
DAPI
E. ch
Beclin1
Merged
Uninfected
DAPI
E. ch
Beclin1
Merged
Infected
DAPI
E. ch
LAMP2
Merged
Uninfected
DAPI
E. ch
LAMP2
Merged
Infected

Fig. S6. Interaction of autophagy regulators and effectors with *E. chaffeensis*. Uninfected and *E. chaffeensis* infected THP-1 cells were examined by immunofluorescence microscopy 30 min post infection. Images show colocalization of *E. chaffeensis* (red) and autophagy effectors LC3 (A) and p62 (B) (green); but not with the autophagy regulators ULK1 (C) and Beclin1 (D) or the lysosomal marker LAMP2 (green). Cells were visualized at 100x magnification. Scale bar represents 10 μm.
